# Food intake and eating behaviour during a real‐life Snack Scenario in childhood obesity—An experiment using a hidden camera

**DOI:** 10.1002/erv.3130

**Published:** 2024-08-09

**Authors:** Isabelle Mack, Jessica Godwin, Bea Klos, Helene Sauer, Alisa Weiland, Björn Horing, Stephan Zipfel, Paul Enck, Nazar Mazurak

**Affiliations:** ^1^ Department of Psychosomatic Medicine and Psychotherapy University Hospital Tübingen Tübingen Germany; ^2^ Department of Systems Neuroscience University Medical Centre Hamburg‐Eppendorf Hamburg Germany

**Keywords:** adolescents, childhood obesity, children, eating behaviour, obesity

## Abstract

**Objective:**

To compare food intake and eating behaviour in children and adolescents with obesity (OBE) undergoing weight loss intervention and normal weight (NW) in a real‐life Snack Scenario.

**Methods:**

Sixty OBE were examined before (T0) and after weight loss (T1) and compared to a single measurement comparison group of 27 NW. Participants watched a 20‐min film and were encouraged to snack from a variety of foods ad libitum. Food intake was measured and eating behaviour assessed via a hidden camera and a validated questionnaire.

**Results:**

The food and energy intake did not differ between NW (155 ± 83 g, 1067 ± 732 kJ) and OBE at T0 (144 ± 106 g, 1088 ± 883 kJ) but increased in OBE at T1 (187 ± 91 g, 1544 ± 845 kJ). Latency of food intake was significantly shorter in NW (0 m:07 s ± 0 m:08 s) compared to OBE (T0: 1 m:11 s ± 2 m:57 s). After weight loss, latency decreased in OBE (0 m:26 s ± 1 m:00 s). NW touched food more often (49 ± 24) than OBE (T0: 29 ± 23), but takes from plate were similar. The questionnaire revealed differences between OBE and NW, not correlating with Snack Scenario observations.

**Conclusion:**

Eating behaviours differed in NW versus OBE at T0 but food intake was similar. Therefore, behaviour while eating may be an underestimated factor in the considerations for childhood obesity.

**Clinical Trial Registration:**

German Clinical Trials Register (DRKS) with the trial number DRKS00005122.

## Highlights

- Childhood obesity is a multifactorial condition influenced by eating behaviour, yet real‐life conditions are rarely studied. There is need for research focusing on eating behaviours, suggesting innovative studies to address and modify behaviours in childhood obesity prevention and treatment.

## 1 INTRODUCTION

Obesity is a growing issue worldwide among children and adolescents (Hall & Kahan, 2018). Although the causes of obesity are multifactorial, an underlying mechanism is an energy imbalance, where caloric intake exceeds expenditure (Hall & Kahan, 2018). Research shows that habitual consumption of large portion sizes and energy‐dense foods leads to excessive energy intake, with children and adolescents with obesity needing larger food volumes to feel satisfied (Benton, 2015; Klos et al., 2023; Mack et al., 2014; Rolls, 2017). Additionally, various eating behaviours have also been associated with obesity in both adults and children (Blundell, 2017; Gahagan, 2012).

Eating behaviours are a combination of inherited and learnt behaviours with cultural, familial and parental influences (Gahagan, 2012; Llewellyn et al., 2008). These behaviours and satiety response change during childhood. Younger children display greater internal appetite and energy regulation, allowing them to compensate for high‐energy snacks by reducing intake at later meals (Obregón et al., 2015). This ability diminishes in older children (Obregón et al., 2015) and may be less effective in children with overweight and obesity compared to those of normal weight (NW) (Munsch et al., 2007).

Children typically prefer energy‐rich, sweet‐flavoured foods (Obregón et al., 2015), likely due to evolutionary adaptations for growth and toxin avoidance. Pleasure from consuming high‐energy density foods is evident even in new‐borns exposed to sweetness (Obregón et al., 2015). Research in adults report that patients with obesity choose more energy‐dense foods (Blundell & Gillett, 2001), however, this preference has not been consistently observed in childhood obesity studies (Kimura et al., 2014; Temple et al., 2008).

The association between eating behaviours involving physical interactions, such as touching food or plate‐to‐mouth actions, and specific food choices and preferences in children and adolescents remains unclear. Nevertheless, visually appealing foods with pleasant textures are more likely to be touched and handled (Lau et al., 1984), and children may interact with foods more frequently when these foods align with their personal preferences.

Eating rate, bite size and the amount of food consumed in a set time are important eating behaviours due to their influence on food intake latency and thus both satiety response and the ability to consume excessive energy (Blundell, 2017). In a video‐monitored, real‐life meal scenario with preschool aged children, Fogel et al. (2017) found that faster eaters consumed 75% more energy than slower eaters, independent of weight status. Other studies have observed significantly faster eating rates in children with overweight or obesity compared to their NW peers (Laessle et al., 2001; Llewellyn et al., 2008). Conversely, addressing these behaviours through interventions aimed at promoting slower eating rates, smaller bite sizes, and mindful eating practices can improve satiety responses and reduce excessive energy intake (Forde et al., 2019).

Currently, data on food intake and eating behaviour in children and adolescents are mainly obtained through questionnaires (Derks et al., 2018; Jendrzyca & Warschburger, 2016; Lansigan et al., 2015) or in experimental settings using plates with concealed digital scales (Laessle et al., 2001). Measuring real‐life food intake and eating behaviour is challenging (Blundell & Gillett, 2001). Of the studies which include the use of video recordings, the focus has been on food amount, energy intake or speed of eating while giving rather limited consideration to the observation of actions and behaviours while eating as latency of food intake, touches of food or plate‐to‐mouth actions (Fagerberg et al., 2019; Fogel et al., 2017). The measurement of specific behaviours exhibited during eating requires creative and complex planning, especially in the case of real‐life scenarios which reduce potential intentional or unintentional changes in eating behaviour, and therefore are rarely investigated.

The aim of this study was to investigate eating behaviour and food choices in children and adolescents with obesity (OBE) before (T0, baseline measurement) and after weight loss (T1, post‐intervention measurement) in comparison to NW peers (NW, single assessment providing a comparative baseline) in a real‐life Snack Scenario and by using a validated questionnaire for eating behaviour. Specifically, we aimed to determine

i) Differences in food intake and eating behaviour: Identify differences in eating rate, bite size, food selection, and satiety response between OBE (pre‐ and post‐intervention) and their NW peers. Understanding these differences can highlight behavioural patterns that contribute to obesity and identify potential intervention points.
ii) Association between real‐life Snack Scenario and questionnaire: Validate self‐reported eating habits by correlating Snack Scenario results with a validated questionnaire for a comprehensive understanding of both observed and self‐reported eating behaviours.
iii) Predictors of weight loss success: Determine if initial behaviours, behaviour changes, or specific patterns predict weight loss success in OBE.

## 2 METHODS

### 2.1 Study population and design

The study presented here was conducted as part of the DROMLIN study (PreDictor Research in Obesity during Medical care ‐ weight Loss in children and adolescents during an INpatient rehabilitation) (Sauer et al., 2014), which aimed to identify predictors for weight loss success in OBE. The study was registered at the German Clinical Trials Register (DRKS) with the trial number DRKS00005122 and the protocol was approved by the Ethics Committee of the University Hospital Tübingen, Germany. Children, adolescents, and their parents were informed about the study and provided written consent prior to inclusion.

Sixty OBE aged 9–17 years, with a body mass index (BMI) above the 90th percentile for their age and sex (Coulthard & Sealy, 2017), were included. They were referred for weight loss hospitalisation to the Children Rehabilitation Hospital for Respiratory Diseases, Allergies, and Psychosomatics in Wangen im Allgäu, Germany, to improve or stabilise their health after exhausting outpatient options. Exclusion criteria included severe psychiatric issues, language or intellectual limitations, type‐1 diabetes, tumours, systemic disorders, or severe heart diseases (Sauer et al., 2014). A multidisciplinary team delivered specialist obesity training as an integral part of the intervention, emphasising a healthy lifestyle including adequate exercise, sufficient sleep, and a balanced diet to promote body health and weight loss. Patients attended weekly small‐group sessions to learn how to prepare and cook food in a training kitchen and participated in ‘shopping training’ in a supermarket. A total of 27 NW matched for age and sex with a BMI between the 10th to 90th BMI percentile were recruited from the University Hospital Tübingen catchment area via a newspaper advertisement and an email to staff at the University Hospital Tübingen and University of Tübingen, Germany.

OBE were tested twice, upon admission (T0) and prior to discharge (T1). NW were tested once and served as a comparison group for OBE T0 measurement. The Snack Scenario took place between 10:45 and 11:15 AM, which was 2.5–3 h after breakfast. The Eating Behaviour and Weight Problems Inventory for Children (EWI‐C) questionnaire was conducted in the afternoon around 3:00 PM (Diehl, 1999). No follow‐up data were gathered.

### 2.2 Assessments

#### 2.2.1 Snack Scenario

Eating behaviour and intake were tested in a Snack Scenario which took place after psychobiological investigations related to the autonomous nervous system by measuring the heart rate variability (Mazurak et al., 2016) and gastric myoelectrical activity (Mack et al., 2014; Weimer et al., 2018). The children and adolescents were invited to relax and watch a film of their choice from a selection of generic children's programmes for 20 min, with casual reference to eat ad libitum from foods provided on a plate within arm's reach. The films were designed to elicit minimal extreme positive or negative emotions and consisted of entertainment and education films. The foods varied in portion size, energy density and were either open or sealed (Table 1). Prior to the experiment, foods were individually weighed on a precision scale before being placed on the plate. The investigator was present in the room at all times, but a dividing wall was placed between the investigator's desk and the unknowing participant. A pocket camcorder (Samsung HMX‐W200, Samsung Electronics (UK) Ltd) recorded the plate of food for the entire Snack Scenario, which lasted 20 min. For privacy policy issues, no patients' faces were filmed. However, filming the plate was sufficient to cover the investigated aspects. The camera was hidden on a shelf adjacent to the table, concealed within a folder allowing the small lens to be hardly visible.

**TABLE 1:** Overview of the offered food items, their amounts, energy density and packaging status.

Food item	Offered amount (g)	Energy density (kJ/100 g)	Energy density classification	Packaging
Apple	∼170	226	Low	Open
Banana	∼130	377	Low	Sealed
Cucumber	∼140	50	Low	Open
Carrot	∼75	138	Low	Open
Pretzel sticks	25	1682	High	Open
Gummy bears	∼50	1473	High	Open
Potato chips	30	2255	High	Sealed
Chocolate bar ‘Kinderriegel’	25	2360	High	Sealed
Shortbread ‘Leibnitz’	25	1807	High	Sealed
Chocolate lentils ‘Smarties’	25	1958	High	Open
Cracker ‘TUC’	15	2033	High	Open

After completion of the experiment, each food item was weighed again, and the amount consumed and corresponding energy intake was calculated and noted. Nutritional data were retrieved from EBISpro 2016 (Stuttgart, Germany). Additionally, the video was analysed and the time points of the touches of food on plate, the type of food and the takes from plate to mouth were counted and documented.

#### 2.2.2 Eating behaviour

Eating behaviour was assessed with the validated German version of EWI‐C consisting of 64 items and 10 subscales (Diehl, 1999). The EWI‐C provides a comprehensive assessment of eating habits and psychological well‐being in relation to diet and weight with higher subscale sum scores corresponding to higher eating behaviour impact. In this analysis, the subscales ‘Hunger level and susceptibility to food cues’, ‘Importance and impact of eating on sense of well‐being’, ‘Eating as a means of coping with emotional stress’, ‘Concerns about eating’, ‘Dietary restraint’, ‘Attitudes towards a healthy nutrition’ and ‘Fear of weight gain’ are reported. The median internal consistency of the 7 subscales in the present sample was *α* = 0.65 [IQR: 0.60–0.71] for OBE at T0, *α* = 0.77 [IQR: 0.72–0.84] for OBE at T1, and *α* = 0.77 [IQR: 0.73–0.82] for NW. Percentile ranks for the values of the subscales were retrieved by sex and age specific norm tables for the questionnaire.

### 2.3 Statistical analysis

The analysis presented here was conducted as part of the DROMLIN study (Sauer et al., 2014), for which the overall sample size was calculated. The aim of the DROMLIN study was to identify predictors that play a role in successful weight loss and body weight maintenance in children and adolescents. For the comparison of eating behaviour between OBE at admission (T0) and NW, a sample size of *n* = 97 allowed to test for medium to large effect sizes (*d* = 0.8) using an unpaired *t*‐test (*α* = 0.05, allocation ratio 1:2) having a power of 0.92, as calculated with G*Power Version 3.1.9.7 (Faul et al., 2009). For the comparison of eating behaviour within the OBE group (T0, T1), robust medium effect sizes (*d* = 0.5) can be measured with a sample size of *n* = 53 using a paired *t*‐test (*α* = 0.05) having a power of 0.95.

Data analysis was performed using IBM SPSS 21 (SPSS Inc.) and MATLAB R2017b (The MathWorks Inc., Natick, MA, USA), with a significance level of < 0.05. To control for multiple testing where appropriate, the *p*‐values were adjusted using false discovery rate (FDR) (Benjamini & Hochberg, 1995) and FDR‐values of < 0.05 were considered as statistically significant. Data is presented as mean ± standard deviation and as median [interquartile range], since not all data were normally distributed. Differences between OBE T0 and NW were calculated using unpaired *t*‐tests (age, weight, BMI z‐score and amount of food intake), Chi^2^ test (sex, number of children and adolescents who took nothing) or Mann‐Whitney‐U‐tests if data were not normally distributed (energy intake, number of touches, number of takes, latency and EWI‐C). Differences between OBE T0 and OBE T1 were analysed with paired *t*‐test (weight, BMI z‐score and amount of food intake) or Wilcoxon signed‐rank test if data were not normally distributed (energy intake, number of touches, number of takes, latency and EWI‐C). OBE were measured twice and NW once, so that NW were not compared to the repeated measurements of OBE (OBE T1).

Subgroup analysis for open, sealed, high‐energy and low‐energy classifications were conducted in the same manner as main analysis. Within‐session changes in eating behaviour were analysed using linear mixed effects models, with adjunct linear mixed effects analyses performed to assess the main contributors to the main results. Spearman correlations were computed to investigate the relationship between eating behaviour and food intake from the experimental setting and EWI‐C subscales.

Multiple linear regression modelling with delta BMI z‐score as the dependent variable and T0 Snack Scenario eating behaviours and EWI‐C subscales as predictors were calculated. Variables were added into regression models in a forward, stepwise manner and Fischer's statistic was used to prove significance of models. Significance was set at *p* < 0.05.

## 3 RESULTS

### 3.1 Study population

Participant characteristics are summarised in Table 2. In OBE, there were 60 participants (m:f 28:32, age 13.0 ± 1.9) and 27 in NW (m:f 15:12, age 12.5 ± 0.9). The length of intervention (inpatient stay) was 38 ± 10 (min‐max: 16–70) days. At T1, seven participants had dropped out so that the longitudinal data refers to a sample of 53.

**TABLE 2:** Characteristics of study participants.

	OBE T0 (*n* = 60)	OBE T1 (*n* = 53)	NW (*n* = 27)	OBE T0 versus NW	OBE T0 versus T1
Sex, m:f	28:32	23:30	15:12	n.s.	n.a.
Age, years	13.0 ± 1.9	13.0 ± 1.9	12.5 ± 0.9	n.s.	n.a.
Range: Min‐max	9–17	9–17	11–14		
Weight, kg	84.0 ± 20.5	80.9 ± 19.9	45.4 ± 8.2	**<0.001**	**<0.001**
Range: Min‐max	51.0–132.0	47.0–128.0	33.8–63.0		
BMI z‐score	2.5 ± 0.6	2.3 ± 0.6	−0.2 ± 0.6	**<0.001**	**<0.001**
Range: Min‐max	1.1–3.7	0.6–3.6	−1.3–1.1		

*Note*: Figures are reported in mean ± SD (age, weight, BMI z‐score) or proportions (sex) for children and adolescents with obesity (OBE) at the beginning (T0) and at the end of inpatient stay (T1) in comparison to normal weight (NW) peers. A *p*‐value < 0.05 is considered as statistically significant, and values are additionally highlighted in bold. Otherwise, data were not significant (n.s.), or comparative statistics was not conducted (n.a.).

Abbreviation: BMI, body mass index.

### 3.2 Food intake and eating behaviour observed during real‐life Snack Scenario

Table 3 provides an overview of the eating behaviour and foods consumed during the 20 min, individually conducted Snack Scenario experiments.

**TABLE 3:** Overview of food consumed during the Snack Scenarios.

	Mean ± SD			Median [IQR]			FDR	FDR
Parameter	OBE T0 (*n* = 60)	OBE T1 (*n* = 53)	NW (*n* = 27)	OBE T0 (*n* = 60)	OBE T1 (*n* = 53)	NW (*n* = 27)	OBE T0 versus NW	OBE T0 versus T1
Food intake, g	144 ± 106	187 ± 91	155 ± 83	125 [75–210]	168 [118–257]	169 [83–214]	0.934	**<0.001**
Energy intake, kJ	1088 ± 883	1544 ± 845	1067 ± 732	841 [456–1665]	1498 [795–2155]	946 [510–1473]	0.934	**<0.001**
Touches of food on plate, n	29 ± 23	37 ± 20	49 ± 24	23 [11–47]	34 [21–52]	47 [28–61]	**0.006**	**0.001**
Takes from plate to mouth, n	17 ± 12	21 ± 11	17 ± 8	16 [6–25]	19 [12–27]	16 [12–22]	0.934	**0.005**
Time of first take from the plate, min:sec	01:11 ± 02:57	00:26 ± 01:00	00:07 ± 00:08	00:15 [00:07–00:48]	00:09 [00:05–00:17]	00:04 [00:02–00:14]	**0.001**	**<0.001**
Number of which took no food	7 out of 60	1 out of 53	0 out of 27	n.a.	n.a.	n.a.	0.397	0.071
**High‐energy foods**								
Food intake, g	51 ± 45	72 ± 44	49 ± 37	41 [16–78]	73 [42–109]	47 [16–70]	0.934	**<0.001**
Energy intake, kJ	950 ± 837	1356 ± 816	929 ± 703	720 [331–1515]	1456 [653–2008]	912 [297–1443]	0.934	**<0.001**
Takes from plate to mouth, n	12 ± 11	14 ± 10	11 ± 6	10 [3–20]	14 [4–21]	11 [6–15]	0.934	**0.036**
**Low‐energy foods**								
Food intake, g	93 ± 85	114 ± 77	106 ± 71	89 [14–138]	115 [47–148]	109 [46–163]	0.785	**0.011**
Energy intake, kJ	134 ± 192	188 ± 201	134 ± 180	26 [4–134]	92 [38–351]	46 [21–151]	0.934	0.071
Takes from plate to mouth, n	4 ± 4	5 ± 4	5 ± 4	4 [0–8]	5 [2–8]	5 [3–8]	0.785	**0.014**
**Open foods**								
Food intake, g	114 ± 89	138 ± 71	121 ± 62	97 [44–163]	130 [83–187]	114 [58–173]	0.934	**0.014**
Energy intake, kJ	720 ± 623	929 ± 569	628 ± 414	540 [138–1054]	849 [490–1435]	611 [276–933]	0.934	**<0.001**
Takes from plate to mouth, n	14 ± 10	17 ± 9	14 ± 7	14 [4–21]	15 [11–23]	14 [10–18]	0.934	**0.034**
**Sealed foods**								
Food intake, g	30 ± 42	49 ± 51	34 ± 49	13 [0–29]	25 [13–80]	16 [0–30]	0.934	**0.001**
Energy intake, kJ	368 ± 385	615 ± 456	435 ± 423	297 [0–590]	590 [297–971]	356 [0–682]	0.934	**<0.001**
Takes from plate to mouth, n	2 ± 5	3 ± 4	2 ± 3	1 [0–2]	2 [1–4]	1 [0–2]	0.785	**0.002**

*Note*: Figures are reported in mean ± SD and Median [IQR] for children and adolescents with obesity (OBE) at the beginning (T0) and at the end of inpatient stay (T1) in comparison to normal weight (NW) peers. FDR <0.05 is considered as statistically significant, and values are additionally highlighted in bold.

Abbreviations: FDR, False Discovery Rate; n.a., not applicable.

#### 3.2.1 Amount and energy of the consumed food

The OBE group consumed significantly more at T1 (187 ± 91 g) than at T0 (144 ± 106 g) (*t*(51) = −4.12, FDR<0.001), thereby also increasing the energy consumed (T0: 1088 ± 883 kJ; T1: 1544 ± 845 kJ) (*Z* = −4.635, FDR<0.001). There was no difference in regard to the amount of food or energy intake consumed between NW (155 ± 83 g, 1067 ± 732 kJ) and OBE at T0.

The food options available were classified into low‐ and high‐energy foods (Table 1). High‐energy food items were chosen at significantly higher proportions than the low‐energy foods across all groups (relative amount of high‐energy foods: OBE T0: 68%; OBE T1: 70%; NW: 70%). No differences between OBE at T0 and NW were found for the amount or energy consumed for low‐ or high‐energy foods. However, OBE significantly increased their intake of both low‐energy and high‐energy foods at T1 compared to T0 (low: *Z* = −2.554, FDR = 0.011; high: *Z* = −4.378, FDR<0.001), resulting in a stable ratio between high‐ and low‐energy food over time.

#### 3.2.2 Influence of packaging on food choice

Open food options were chosen at significantly higher proportions than sealed foods by both OBE and NW, with no differences between groups (relative amount of opened foods: OBE T0: 92%; OBE T1: 91%; NW: 92%). The consumption of sealed foods and open foods both increased at T1 compared to T0 (open: *Z* = −2.466, FDR = 0.014, sealed: *Z* = −3.265, FDR = 0.001), resulting in the ratio of open to sealed foods to be unchanged.

#### 3.2.3 Latency of food intake

**FIGURE 1:**
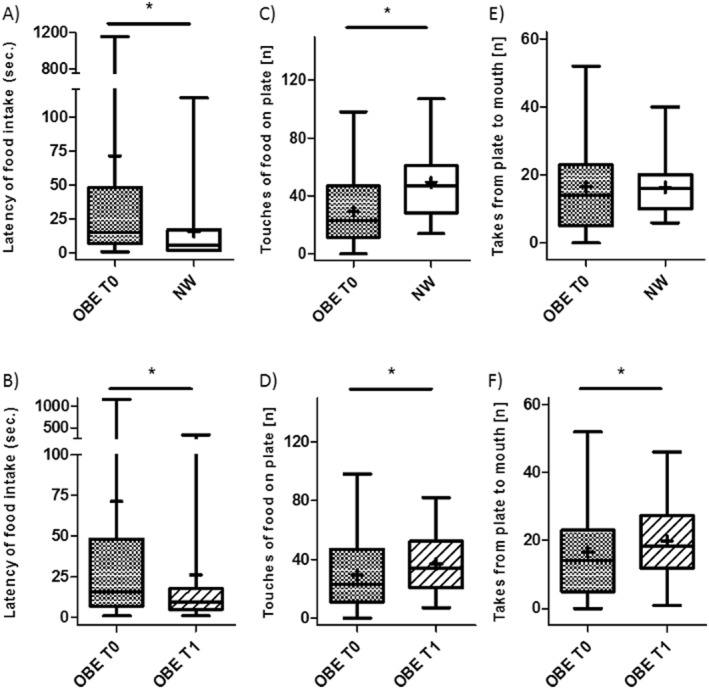
Eating behaviour assessed by experiment. Legend: Time until first food intake (latency, a, b), number of food touches on the plate (c, d), and takes from plate to mouth (e, f) in children and adolescents with obesity (OBE) at the beginning (T0) and at the end of inpatient stay (T1) in comparison to normal weight (NW) peers.

The latency until initial food intake in minutes decreased from T0 (1 m:11 s ± 2 m:57 s; range: 0 m:01 s–19 m:19 s) to T1 (0 m:26 s ± 1 m:00 s; range: 0 m:01 s–5 m:38 s) (*Z* = −3.652, FDR<0.001) in OBE but was longer compared to NW (0 m:07 s ± 0 m:08 s; range: 0 m:02 s–0 m:32 s) (*U* = 332.5, FDR = 0.001), as displayed in Figure 1a,b. All NW took food from the plate during the study, whereas 7/60 OBE at T0 and 1/52 at T1 did not take any food.

#### 3.2.4 Number of touches and takes from plate

The number of times the children and adolescents touched food on the plate (regardless of whether they ate the food) and the number of takes from plate to mouth were counted (Figure 1c–f). The number of touches of food on the plate was highest in NW (49 ± 24) compared to OBE T0 (29 ± 23, *U* = 433, FDR = 0.006), however, the number of takes from plate to mouth did not differ between NW (17 ± 8) and OBE at either time point (T0: 17 ± 12; T1: 21 ± 11). Comparison of OBE at T0 and T1 showed an increase in the total number of food touches (*Z* = −3.515, FDR<0.001) and the number of takes from plate to mouth (*Z* = −2.935, FDR = 0.005).

### 3.3 Within‐experiment time interval breakdown

**FIGURE 2:**
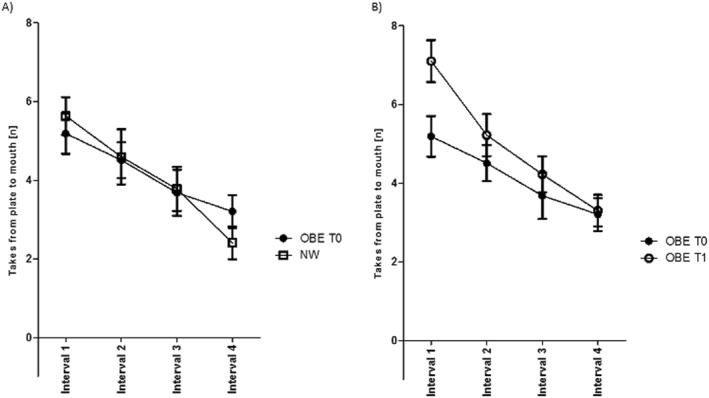
Number of takes from plate to mouth at each 5‐min interval for children and adolescents with obesity (OBE) at the beginning (T0) versus normal weight (NW) and OBE T0 versus at the end of inpatient stay (T1) (b). Data shown as mean ± SD. OBE T0 deceleration slope did not differ from NW, whereas OBE T1 had an accelerated decline compared to T0.

To identify within‐session changes in eating behaviour, we segmented the 20‐min‐long sessions into four 5‐min intervals. In all groups, most plate‐to‐mouth actions occurred in the initial interval, reflecting the impact of satiety. OBE T0 and NW demonstrated a steady decline in plate‐to‐mouth actions (0.9 less every 5 min, FDR<0.001) (Figure 2a). On average, plate‐to‐mouth actions increased from T0 to T1 (0.9 more occasions at T1, FDR = 0.011), with a significant increase during the first interval followed by an accelerated decrease (at T1, there were 1.2 fewer occasions every 5 min) to align with a similar number of takes during interval 4 at T0 (interaction segment*session, FDR = 0.010) (Figure 2b).

Additional analyses were performed to understand within‐session changes in food choices, considering open versus sealed and low‐ versus high‐energy items. This showed that the takes from plate to mouth for open foods followed the same pattern as total food consumed, with no difference between OBE T0 and NW (FDR = 0.98), but a linear decrease across groups (FDR<0.001). No difference was also found between OBE at T0 and T1 (FDR = 0.07) (accelerated decrease at T1 with FDR = 0.016). Sealed foods were rarely selected at each interval; however were consistently chosen. There was a trend for high‐energy foods to be taken at a higher rate during the first interval at T1 than at T0, however this was not significant. Lastly, OBE at T1 displayed an accelerated decline at each interval for high‐energy foods compared to T0 (interaction segment*session, FDR = 0.026).

#### 3.3.1 Influence of secondary variables

To assess the impact of secondary variables, we repeated the main analyses with the inclusion of several covariates. These were the amount and energy intake of the breakfast consumed approximately 3 h before the Snack Scenario (Weimer et al., 2018), the stress rating from the stress test completed immediately before the Snack Scenario (Mazurak et al., 2016), and information collected regarding the participant's taste preferences for the 4 tastes (sweet, sour, salty, bitter) (Sauer et al., 2017). Inclusion of these covariates did not alter the above findings (data not shown).

### 3.4 Eating behaviour assessed by a questionnaire

Eating behaviour subscales from the EWI‐C are presented in Table 4. Comparing the OBE T0 with NW, the OBE group was significantly more likely to use eating as a means of coping with emotional stress (*U* = 480, FDR = 0.004), have concerns about eating and weight gain (*U* = −226, FDR<0.001), and express dietary restraint (*U* = 387.5, FDR<0.001). Over the course of intervention, the OBE results remained stable, with an improved score only reported for concerns about eating (*Z* = −7.073, FDR = 0.034). This indicates a significant reduction in these concerns, suggesting that the OBE are less burdened by weight and eating problems at T1. EWI‐C subscales did not correlate with the eating behaviours observed during the Snack Scenarios after alpha correction of the *p*‐values.

**TABLE 4:** Eating behaviour as measured by EWI‐C subscales.

	OBE T0 (*n* = 60)	OBE T1 (*n* = 53)	NW (*n* = 27)	FDR	FDR
Subscale				OBE T0 versus NW	OBE T0 versus T1
Hunger level and susceptibility of food cues	47 [21–67]	40 [16–63]	41 [16–62]	0.382	0.089
Importance and impact of eating on sense of well‐being	42 [24–62]	36 [19–50]	48 [29–59]	0.851	0.089
Eating as a means of coping with emotional stress	71 [41–89]	71 [36–89]	41 [22–71]	**0.004**	0.147
Concerns about eating	90 [78–96]	84 [78–90]	25 [24–53]	**<0.001**	**0.034**
Dietary restraint	65 [47–82]	67 [57–84]	38 [22–59]	**<0.001**	0.389
Attitudes towards a healthy nutrition	75 [52–81]	75 [52–94]	85 [39–94]	0.208	0.389
Fear of weight gain	79 [46–92]	70 [52–87]	30 [27–46]	**<0.001**	0.669

*Note*: Eating behaviour is measured for children and adolescents with obesity (OBE) at the beginning (T0) and at the end of inpatient stay (T1) in comparison to normal weight (NW) peers. Figures reported as Median [IQR]. FDR <0.05 is considered as statistically significant, and values are additionally highlighted in bold.

Abbreviation: EWI‐C, Eating Behaviour and Weight Problems Inventory for Children; FDR, false discovery rate.

### 3.5 Prediction of weight loss outcomes

Regression analysis was conducted to assess the predictive power of the observed eating behaviours, amount and energy consumed, and EWI‐C subscales at baseline on weight loss achieved between T0 and T1 for the OBE group (delta BMI z‐score). The amount of food consumed by OBE at T0 was identified as a weak predictor of weight loss (*R* ^2^ = 0.106, *F* = 7.176, *p* = 0.01) with higher amounts corresponding to smaller weight loss; however, baseline eating behaviour measures did not predict the outcome.

## 4 DISCUSSION

This study reports eating behaviour observations in a real‐life, ad libitum Snack Scenario and an eating behaviour questionnaire. Our findings show that the OBE group consumed the same amount of food, the same total energy and had the same number of takes from plate to mouth as their peers (NW), however displayed significantly different eating behaviours for latency and number of touches on plate.

Despite no difference in the total amount or energy consumed, NW group had in comparison to OBE a quicker average latency before taking food, while all NW participants consumed food during the Snack Scenario. This identifies higher self‐restraint in the OBE group, as the Snack Scenario was conducted 2.5–3 h after breakfast when a snack might be expected. This was additionally apparent when looking at the EWI‐C scores with OBE T0 reporting significantly higher dietary restraint and concerns about eating compared to NW. These findings, along with the higher prevalence of emotional eating, align with the literature for children and adolescents with overweight and obesity as assessed by questionnaires (Byrne et al., 2019; Derks et al., 2018; Hofmann et al., 2015; Temple et al., 2008).

Another difference between the OBE and NW groups was the number of touches of food on plate. Although there was no significant difference between the number of takes from plate to mouth between OBE and NW, and consequently similar food intakes, the NW touched the food much more often than the OBE. This may indicate NW children's greater comfort in exploring different foods and playfully interacting with them. Developing a healthy curiosity about different foods' tactile qualities is crucial for children's food acceptance, particularly for low‐energy foods (fruits and vegetables) (Coulthard & Sealy, 2017; Coulthard & Thakker, 2015). Increased tactile enjoyment and playful food exploration have been associated with lower food avoidance and neophobia in preschool children (Coulthard & Thakker, 2015). Although neophobia and fussy eating are fairly common in young children, its prevalence may continue into adolescence and adulthood, linked to lower diet quality and metabolic risk factors (Sarin et al., 2019). However, the relationship between food avoidance and weight status in childhood and adolescence remains unclear (Brown et al., 2016).

Differences were observed for the food intake and eating behaviours of OBE between T0 and T1, with increased intake, reduced latency and more touches and takes from plate. The reduction in BMI z‐score during intervention suggests that the increased energy intake at T1 may be a compensatory response to counteract weight loss. Another explanation could be the familiarity with the Snack Scenario in their testing schedule, leading to anticipatory food choices. The improved score on the EWI‐C subscale ‘concerns about eating’ at T1 indicates reduced weight and eating problems in OBE. Predictor analysis results exclude this as a reason for the increased energy intake at T1.

Satiety effects were seen in all Snack Scenario groups (NW, OBE T0 and OBE T1), with the number of takes from plate to mouth declining with each 5‐min interval. The observed deceleration in food intake over a meal can help prevent passive overconsumption (Zandian et al., 2009). Llewellyn et al. noted a similar weight‐independent trend in children during a video recorded family meal (Llewellyn et al., 2008).

The total amount of food weakly predicted weight loss success in the OBE group. Importantly, the average amount of food consumed by OBE at T0 did not differ from the NW group. However, this weak prediction might reflect the benefits of a moderate mindset for weight loss and maintenance (Hall & Kahan, 2018). Eating behaviours are not commonly explored as weight change predictors, instead assessed observationally. Our study found that eating behaviours or total energy intake were not predictive of weight change, aligning with recent findings in children (Boutelle et al., 2019; Kelly et al., 2015).

Deviations from normal eating behaviours and their connection to weight status are well‐documented, especially in extreme cases like anorexia nervosa and binge eating disorder (Baldofski et al., 2015; Gianini et al., 2015). However, the nuances of different eating behaviours in the field of obesity are unexplored, with its implications for weight management approaches unclear (Fogel et al., 2017; Llewellyn et al., 2008). The school environment might be crucial for studying eating behaviour in children and adolescents, as about 40% total daily energy is consumed during school hours (O’Halloran et al., 2020). A recent review of school food environments assessed multiple aspects including the physical environment, compliance with guidelines and food options available, however aspects relating to eating behaviour were not assessed (O’Halloran et al., 2020). Therefore, the influence of the school food environment on the school children's and adolescents' eating behaviours remains unknown.

The study has several strengths and limitations. A limitation is that NW were tested once, whereas OBE were tested twice, making it challenging to account for familiarity's impact on food intake upon repeat measures. Additionally, the current focus on eating behaviour did not extend to rate of eating/bite size as sometimes investigated (Fogel et al., 2017; Llewellyn et al., 2008), and did not assess eating in the absence of hunger as conducted in other studies (Fogel et al., 2018). Finally, the present Snack Scenario provides a snapshot into the eating behaviours of children and adolescents, but cannot fully capture everyday eating complexity. Nevertheless, our results provide the insight that eating behaviour is different between groups, even without a net difference in intake. Possibly, a snapshot of eating behaviour might provide more insights into overall dietary behaviour than observations or records of food intake (Collins et al., 2010; Walker et al., 2018), though this remains speculative.

A clear strength lies in minimising external influences on eating behaviours using hidden cameras to capture real‐life behaviours. The food was provided at a typical snack time of day and included a combination of common child snack items, therefore promoting intake at this time and limiting the impact of unfamiliarity on food choices. Participants completed the Snack Scenarios individually, reducing peer influence and social desirability effects. Eating behaviour in children and adolescents has been frequently observed in group settings (Fagerberg et al., 2019; Warkentin et al., 2020), however research in adolescents show that they alter their food choices depending on the social environment (Contento et al., 2006; Martin et al., 2018).

This study highlights the significance of eating behaviours in children and adolescents with obesity. It underscores the need for future research to examine these behaviours and develop effective prevention and treatment strategies. Future studies should explore the relationships between various eating behaviours to identify predictors of weight loss and maintenance, potentially uncovering key factors for successful weight management in children and adolescents with obesity.

## 5 CONCLUSION

The outcomes of the present study show that OBE display different eating behaviours than NW children in a real‐life Snack Scenario but consumed a similar energy intake. This finding is mirrored by scores in the EWI‐C questionnaire. During weight loss intervention, changes in parameters related to both intake and eating behaviour variables may suggest a compensatory mechanism following significant BMI reduction, possibly influenced by familiarity with the Snack Scenario. Although the Snack Scenarios only provided a snapshot into the participants' eating behaviours, these findings and continued research provide important insight into not only what we eat, but how we eat. The future of childhood obesity research requires clever and well‐designed studies to examine behaviours specific to eating occasions and assess strategies for addressing and modifying these behaviours in the context of the prevention and treatment of childhood obesity.

## AUTHOR CONTRIBUTIONS

Isabelle Mack analysed the data. All authors were involved in writing the paper and had final approval of the submitted and published versions.

## CONFLICT OF INTEREST STATEMENT

The authors declare no conflict of interest.

## Data Availability

Data supporting the findings of this study are available from the corresponding author, I.M., upon reasonable request.
